# The Relationship between CrossFit^®^ Performance and Laboratory-Based Measurements of Fitness

**DOI:** 10.3390/sports8080112

**Published:** 2020-08-11

**Authors:** Elisabeth K. Zeitz, Lena F. Cook, Joshua D. Dexheimer, Srdjan Lemez, Whitney D. Leyva, Immanuel Y. Terbio, Justin R. Tran, Edward Jo

**Affiliations:** 1 Human Performance Research Laboratory, Department of Kinesiology and Health Promotion, California State University Pomona, Pomona, CA 92805, USA; ekatzeitz@gmail.com (E.K.Z.); lfcook@cpp.edu (L.F.C.); slemez@cpp.edu (S.L.); whitleyva@gmail.com (W.D.L.); immanuelterbio@gmail.com (I.Y.T.); justinrtran@gmail.com (J.R.T.); 2 Department of Kinesiology, Azusa Pacific University, Azusa, CA 91702, USA; jdexheimer@apu.edu

**Keywords:** high-intensity functional training, aerobic fitness, body composition

## Abstract

To date, research has examined the physiological determinants of performance in standardized CrossFit^®^ (CF) workouts but not without the influence of CF familiarity. Therefore, the purpose of this present study was to examine the predictive value of aerobic fitness, body composition, and total body strength on performance of two standardized CF workouts in CF-naïve participants. Twenty-two recreationally trained individuals (males = 13, females = 9) underwent assessments of peak oxygen consumption (VO_2_ peak), ventilatory thresholds, body composition, and one repetition maximum tests for the back squat, deadlift, and overhead press in which the sum equaled the CF Total. Participants also performed two CF workouts: a scaled version of the CF Open workout 19.1 and a modified version of the CF Benchmark workout Fran to determine scores based on total repetitions completed and time-to-completion, respectively. Simple Pearson’s r correlations were used to determine the relationships between CF performance variables (19.1 and modified Fran) and the independent variables. A forward stepwise multiple linear regression analysis was performed and significant variables that survived the regression analysis were used to create a predictive model of CF performance. Absolute VO_2_ peak was a significant predictor of 19.1 performance, explaining 39% of its variance (adjusted R^2^ = 0.39, *p* = 0.002). For modified Fran, CF Total was a significant predictor and explained 33% of the variance in performance (adjusted R^2^ = 0.33, *p* = 0.005). These results suggest, without any influence of CF familiarity or experience, that performance in these two CF workouts could be predicted by distinct laboratory-based measurements of fitness.

## 1. Introduction

CrossFit^®^ (CF; CrossFit Inc., Washington, DC, USA) is a branded fitness program established in 2000 and is characterized by “constantly varied, high intensity, functional movements aimed to elicit a change in work capacity over time” [1]. CF is often described as high-intensity functional training (HIFT) incorporating multimodal compound movements performed with relatively high intensity for a predetermined timeframe or volume [2]. The exponential growth of CF is unprecedented and signified by over 15,000 CF affiliated gyms worldwide and its global recognition as the “sport of fitness” [3]. With that, CF has evolved into a worldwide competitive sport highlighted by the annual CrossFit Games^®^ which includes the top performers in the preceding CF Open.

Programmed bouts of CF, known as the Workout of the Day (WOD), are constantly varied across each session and typically includes an element of either gymnastics (e.g., handstands or pull-ups), weightlifting (e.g., deadlifts or Olympic lifts), aerobic conditioning (e.g., running, rowing, or cycling), or a combination of two or three of each element [1]. Workouts/WODs are completed either with a time demand in which participants are given a specific timeframe to complete as many repetitions as possible (AMRAP) of the selected movements, or with a task demand in which the given exercise tasks must be completed in the shortest amount of time possible. The WODs are to be performed with maximal effort as quickly and efficiently as possible without sacrificing form [1]. Popular WODs include Fran, Grace, and Cindy. Fran is a time-scored workout that consists of three rounds of a descending 21–15–9 repetition scheme of thrusters (front squat to press overhead) and pull-ups. Grace consists of 30 clean and jerks for time, and Cindy is a 20 min workout that requires AMRAP of five pull-ups, 10 push-ups, and 15 air squats to be completed within the allotted time period. Also, WODs may be specially designed for the CF Open such as the 19.1 which involves a 15 min AMRAP-style workout consisting of a couplet of 19 wall balls and a bout of stationary rowing for 19 calories as indicated by a microcomputer interfaced to a standard CF-approved rower.

As a means to assess fitness and changes in work capacity over time, CF has implemented standardized workouts called Benchmark WODs that are distinguished by their specific exercises, structure, physical demands, and scoring mechanism which includes total repetitions within a given timeframe or time to completion of a target number of repetitions [4]. It is claimed that performance in Benchmark WODs is reflective of a participant’s health and fitness status [1]. Although this claim is generally anecdotal, prior empirical evidence demonstrates positive correlations between performance on select Benchmark WODs and various measures of fitness and health [5,6,7]. From a competitive CF standpoint, Benchmark WOD performance has also shown to be directly correlated to and indicative of performance in the CF Open providing athletes a means to assess their potential as a competitor [8]. By better understanding the role of specific performance and health variables in CF, athletes would be capable of identifying the physical and physiological limitations to CF performance and success.

A body of CF research has been dedicated to discovering the predictive strength of various laboratory-based human health and performance variables on Benchmark WOD performance. Butcher et al. [6] found that performance on the Benchmark WODs, Fran, Grace, and Cindy were not indicated by aerobic capacity or peak anaerobic power, and Fran and Grace performance was directly correlated with anaerobic threshold (AT) and total body strength. Strength measures, including back squat one-repetition maximum (1RM) and CF Total, a sum of back squat, deadlift, and overhead press 1RM, were predictive of performance in Fran [7,9] and positively related to ranking in the 2016 CF Open [8]. In a separate study by Bellar et al. [5], CF experience (CF-athlete vs. CF-naïve) was the strongest predictor of performance in an AMRAP-style CF workout versus physiological performance variables such as aerobic capacity and peak power. Thus, it appears there is influence of CF experience or familiarity on the predictive strength of physiological performance variables, such as aerobic capacity. Hence, there is merit in investigating the predictive value of laboratory-based measures of fitness and performance in CF-naïve participants to better understand whether these physiological variables truly predict CF performance without interference of CF familiarity. Therefore, the purpose of the current study was to examine, in CF-naïve participants, the predictive strength of laboratory-based measurements of aerobic fitness, body composition, and total body strength on performance of two distinct CF WODs: 19.1 and modified Fran. It was hypothesized that: (1) maximal oxygen consumption, ventilatory thresholds, muscular strength, and body composition would be correlated to performance in 19.1 and modified Fran; and (2) these variables could partially predict performance in those CF WODs.

## 2. Materials and Methods

### 2.1. Experimental Design

This was a descriptive observational design study of a random sample of CF-naïve but recreationally trained individuals to examine the predictive strength of laboratory-based measures of fitness on CF WOD performance. During Visit 1, participants arrived at the Human Performance Research Laboratory at California State Polytechnic University, Pomona and signed an Informed Consent Form followed by an exercise and health history questionnaire and Physical Activity Readiness Questionnaire (PAR-Q) [10]. Afterwards, participants underwent anthropometric measurements which included body height and weight, followed by body composition assessment via multi-frequency bioelectrical impedance analysis (BIA). Next, participants performed a graded maximal treadmill test to determine peak oxygen consumption rate (VO_2_ peak), ventilatory threshold 1 (VT1), and ventilatory threshold 2 (VT2) using indirect calorimetry and breath-by-breathe analysis.

Approximately 48 h later, participants returned to the laboratory for Visit 2 and performed an assessment of total strength, also known as “CF Total”, which included a structured warm-up to familiarize participants with the movements employed in the WODs, then 5 min of a self-selected warm-up, followed by 1RM tests for the back squat, overhead press, and deadlift, in the listed order.

During Visits 3 and 4 (non-consecutive days), participants performed either the modified 19.1 or Fran CF WOD in a randomized and counterbalanced order. 19.1 score was measured by total repetitions performed while the modified Fran score was determined by time-to-completion. All WODs were supervised by a CF Level 1 Trainer who recorded performance data for each participant and ensured workout standards were met. All volunteers signed an informed consent form prior to participation, and this study was approved by the Institutional Review Board at California State Polytechnic University, Pomona (IRB 19-247).

### 2.2. Participants

Twenty-two healthy male (*n* = 13) and female (*n* = 9) participants were recruited for this study. Descriptive measures can be found in Table 1. Participants met the following inclusion criteria to participate: (1) at least 18 years of age, (2) engaged in resistance and/or aerobic training 2–3 days a week for the past year, and (3) naïve to CF (no participation in CF training for the past year). Although by definition all participants were naïve to CF, three of the 22 participants partook in CrossFit^®^ within the past three years, but not within the past year, deeming them eligible for participation. Participants were excluded from participation if they reported a medical or surgical history that would contraindicate the experimental protocol and/or confound the interpretation of results. These included, but were not limited to, (1) cardiovascular, pulmonary, metabolic, or renal diseases; (2) hypertension; (3) smoking; and (4) use of any medication or drugs, including those with cardiovascular, pulmonary, hyperlipidemic, hypoglycemic, hypertensive, and/or birth control effects. In addition, participants were told they would be excluded if they utilized dietary ergogenic aids daily within six weeks prior to the study. Daily use of nutritive supplements (e.g., whey protein or multivitamins) did not call for exclusion. Participants were asked to maintain their normal physical activity/exercise levels and dietary intake during the timespan of the study.

### 2.3. Laboratory Testing Protocols

#### 2.3.1. Body Composition

Body composition was measured via multi-frequency BIA (InBody 720 system, Inbody USA, Cerritos, CA, USA). Before the measurement, participants’ palms and feet were cleansed of any residual electrolytes from bodily fluids. Participants stood on the InBody 720 platform with the soles of their feet in contact with the interfaced electrodes. The instrument derived the participants’ body mass, and their age, sex, and height were manually inputted. Participants then grasped the handheld electrodes with arms fully extended and abducted about 20 degrees. Analysis was performed with the participant motionless. Prior test–retest reliability assessment indicated the following: fat mass (Intraclass Correlation (ICC) = 0.998), fat free mass (ICC = 1.00), and body fat percentage (ICC = 0.995) [11].

#### 2.3.2. Aerobic Fitness

The measurement of VO_2_ peak, VT1, and VT2 was performed using a maximal graded treadmill exercise test protocol and an open-circuit indirect calorimeter metabolic cart (Quark CPET; Cosmed USA Inc; Concord, CA, USA). The assessment was administered in a thermo-neutral (~24 °C) room. For the VO_2_ peak assessment, participants reported to the laboratory following at least 8 h of no strenuous activity and rested quietly in a seated position for 10 min before testing. During this period, blood pressure and resting heart rate were measured for precaution to ensure the participant was under appropriate cardiovascular conditions prior to the test. The participant was then fitted with a rubber ventilated mask covering the nose and mouth. The mask was interfaced to the indirect calorimeter/metabolic cart and breath-by-breath analysis was implemented. Participants sat for 1–2 min followed by a 3 min low intensity walk on a treadmill as a warm-up and to ensure normal responses by the metabolic cart. Following 3 min of warm-up, the participants underwent a series of 3 min stages with increasing intensity until peak oxygen consumption was achieved. After each stage, the participant was asked to report on a 6–20 Borg Rating of Perceived Exertion (RPE) scale with 6 indicating “no effort” and 20 representing “maximum effort” [12]. Also, after each stage, participants were asked to provide a hand signal that corresponded to their level of exertion (thumbs up = proceed to next stage, thumbs down = indication of exhaustion approaching, cutting motion = stop the test). Participants were verbally encouraged to reach maximal effort. The test was terminated at volitional failure. Peak oxygen consumption was determined as the highest VO_2_ obtained during the test and was confirmed by a respiratory exchange ratio of 1.1 or greater and achievement of VT2 (also known as respiratory compensation) as defined below [13]. Ventilatory threshold 1 was determined using the criteria of an increase in both ventilatory equivalent of oxygen (VE/VO_2_) and end-tidal pressure of oxygen (PETO2) with no concomitant increase in ventilatory equivalent of carbon dioxide (VE/VCO2). Ventilatory threshold 2 was determined using the criteria of an increase in both the VE/VO_2_ and VE/VCO_2_ and a decrease in PETCO2 [14].

#### 2.3.3. Familiarization and CrossFit^®^ Total

Participants were instructed to complete 2 min of rowing on a rowing ergometer (Model D, Concept 2, Morrisville, VT, USA), followed by one round of: five repetitions of wall balls, five repetitions of thrusters, and five burpees over the barbell. These procedures ensured participants were physically capable of performing the movements employed in the WOD protocols described below. Afterwards, participants performed 5 min of a self-selected warm-up prior to strength testing.

Participants were then tested for 1RM of the back squat, overhead press, and deadlift exercise, the sum of which equal the CF Total. Participants performed 8–10 repetitions with 50% of their estimated 1RM for each lift, followed by five repetitions at 75% 1RM, three repetitions at 85% 1RM (with 1–2 min of rest in between), and then were given 3–5 separate attempts to determine their 1RM for each lift. Between each attempt, participants were given 3 min to rest, and 5 min of rest between each different lift. 

### 2.4. WOD Protocols

#### 2.4.1. The 19.1

The 19.1 protocol was a scaled version of the first WOD from the 2019 CF Open which was a 15 min AMRAP-style workout consisting of a couplet of 19 wall balls (males used 6.4kg, females used 4.5 kg) and a bout of stationary rowing for 19 calories as indicated by the microcomputer interfaced to the rower (Model D, Concept 2, Morrisville, VT, USA). Performance was scored by the total number of repetitions completed in the 15 min time period.

#### 2.4.2. Modified Fran

The modified Fran was a variation of the Benchmark WOD Fran and consisted of three rounds (Round 1 = 21 repetitions, Round 2 = 15 repetitions, Round 3 = 9 repetitions) of thrusters (a front squat completed with an overhead press, 20 kg barbell for males, 16 kg barbell for females) and burpees over the barbell. Performance was measured by time to completion. A higher score was indicated by a lower time to completion.

### 2.5. Statistical Analysis

Preliminary analyses were performed to confirm that there was no violation of assumptions of normality of residuals (Kolmogorov–Smirnov and P–P plot), linearity (scatterplots), multicollinearity (tolerance and variance inflation factor), and homoscedasticity (scatterplot of standardized residuals and predicted values). Data were inspected for outliers using Cook’s distance analysis. Simple Pearson’s r correlations were used to determine the relationship between CF performance variables (19.1 and Fran) and the independent variables. The magnitude of the correlations was classified as follows: r ≤ 0.1 trivial; 0.1 < r ≤ 0.3 small; 0.3 < r ≤ 0.5 moderate; 0.5 < r ≤ 0.7 large; 0.7 < r ≤ 0.9 very large; r > 0.9 almost perfect [15]. A forward stepwise multiple linear regression analysis was utilized for each WOD. Adjusted correlation of determination (R^2^) was used to determine the predictive power of the models and calculated by the following formula: Adjusted R^2^ = 1 − (1 − R^2^) (N − 1)/N – p – 1; where R^2^ = sample R-squared, *p* = number of predictors, and *n* = total sample size. Each CF variable was also incorporated as independent variables in the stepwise regression analysis of the other CF variable. Data are reported as means and standard deviations. The alpha level was set a priori at 0.05. Data analyses were performed on SPSS Version 25 (IBM, Armonk, NY, USA).

## 3. Results

To satisfy the assumption of multicollinearity, all independent variables except for absolute VO_2_ peak, CF Total, BF%, age, and sex were excluded from the stepwise multiple linear regression analysis. Two outliers for modified Fran performance were removed pairwise from the analyses which in turn altered the Cook’s distance to meet the criteria of <1. Table 1 displays the mean values for all independent and dependent variables for the total participant pool and by sex.

**Figure 1:**
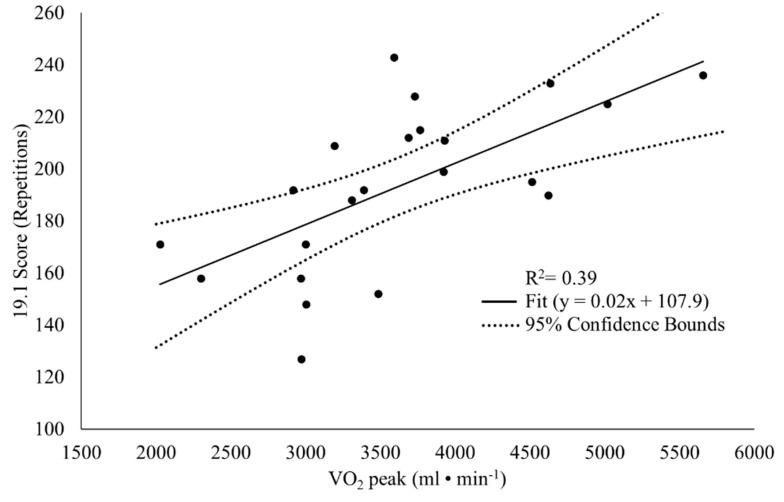
19.1 score as a function of VO_2_ peak.

**Figure 2:**
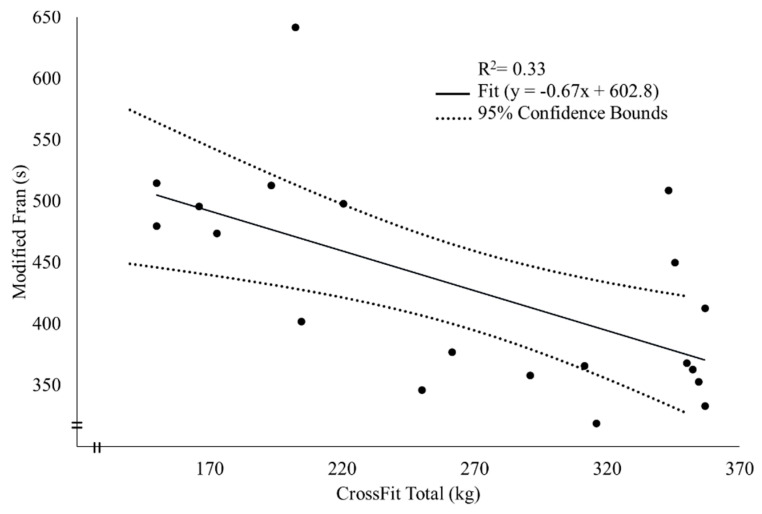
Modified Fran time as a function of CrossFit^®^ Total.

There was a significant positive correlation between 19.1 score and back squat (*p* = 0.01, r = 0.58, large), overhead press (*p* = 0.004, r = 0.59, large), and deadlift (*p* = 0.002, r = 0.62, large) 1RM, CF Total (*p* = 0.002, r = 0.62, large), relative CF Total (*p* = 0.03, r = 0.46, moderate), body mass (*p* = 0.01, r = 0.53, large), absolute VO_2_ peak (*p* = 0.001, r = 0.65, large), relative VO_2_ peak (*p* = 0.02, r = 0.48, moderate), and absolute VO_2_ at VT1 (*p* = 0.01, r = 0.56, large) and VT2 (*p* = 0.002, r = 0.61, large; Table 2). For 19.1 performance, as measured by total repetitions, stepwise multiple linear regression resulted in a significant model (adjusted R^2^ = 0.39, *p* = 0.002) in which absolute VO_2_ peak survived as the sole predictor variable (β = 0.65, *p* = 0.002; Figure 1, Table 3). All other variables had no further impact on the prediction of 19.1 performance. There was a significant negative correlation between modified Fran time-to-completion and back squat (*p* = 0.01, r = −0.58, large), overhead press (*p* = 0.003, r = −0.63, large), and deadlift (*p* = 0.01, r = −0.57, large) 1RM, CF Total (*p* = 0.01, r = −0.61, large), and relative CF Total (*p* = 0.004, r = −0.62, large). For modified Fran performance, as measured by time to completion, a significant predictive model was found (adjusted R^2^= 0.33, *p*= 0.005) in which CF Total was the sole predictor (β = −0.61, *p* = 0.005; Figure 2, Table 4). All other variables had no further influence on the prediction of modified Fran performance.

## 4. Discussion

The overarching objective of this investigation was to determine the predictive value of aerobic fitness, body composition, and total body strength on performance of two CF WODs, 19.1 and the modified Fran in CF-naïve participants. As an executive summary of findings, all strength variables were positively correlated (moderate to strong) to performance in 19.1 and modified Fran (negatively correlated with time-to-completion), indicating greater CF performance with greater strength and vice versa. As for body composition variables, body fat percentage was negatively correlated (moderate) only to 19.1 performance. Overall, measures of aerobic fitness, which included VO_2_ peak, VT1, and VT2, were positively correlated (moderate to strong) with 19.1 performance, while no correlations were found with modified Fran performance. Linear regression analysis showed that absolute VO_2_ peak was the sole indicator of 19.1 performance as it explained 39% of the variance in total repetitions achieved. The CF Total (CF-specific measurement of total body strength) was the only significant predictor of the modified Fran performance and explained 33% of the variance in the modified Fran time-to-completion.

To our knowledge, the present investigation was the first to examine the predictive strengths of laboratory-based fitness variables without influence of CF familiarity/history by recruiting only those naïve to CF. The findings of Martinez-Gomez et al. [16] and Bellar et al. [5] are especially relevant to the present results for 19.1. The 19.1 workout was of interest for the present study because it is a globally standardized WOD as found in the 2019 CF Open, and it incorporates basic exercises that participants could familiarize with in a relatively short period (wall balls and rowing). Martinez-Gomez et al. [16] examined physiological predictors of performance in 2019 CF Open WODs which included 19.1. Results indicated that VO_2_ max was positively correlated (r = 0.63; *p* < 0.05) with 19.1 performance and indicative of overall CF Open performance. Additionally, Bellar et al. [5] found that relative VO_2_ max positively correlated (r = 0.65, *p* = 0.03) with performance in a similar AMRAP-style workout that included wall balls in both CF-naïve and CF-trained individuals. These results agree with the findings of the present study in which absolute VO_2_ peak was positively and strongly related to 19.1 performance. Peak oxygen consumption rate was also the sole significant indicator of 19.1. Because VT1 and VT2 were strongly correlated with VO_2_ peak and thus, collinear, VT1 and VT2 were removed from the regression analysis. However, given the strong correlation between each of these two variables and VO_2_ peak, it can be assumed that VTs would also be indicators of 19.1 performance. Aerobic capacity (as quantified by VO_2_ max/peak) and anaerobic thresholds as measured for instance by VTs and lactate threshold are variables that majorly constitute aerobic power or fitness [17,18]. Given the metabolic demands of 19.1, it is apparent that aerobic power would be an important determinant of performance. To expound further, a critical limitation to performance in metabolically challenging exercise such as the 19.1 is acute muscular fatigue as defined as an immediate inhibition of skeletal muscle contractility due to an acute localized hypoxic, anaerobic stress [19,20,21,22]. With this in mind, it was anticipated that relatively higher levels of aerobic fitness, as determined by VT and VO_2_ max/peak, would be associated with superior performance in 19.1 because the aerobic system could fulfill the greater portion of energy demand at higher intensities without relying on anaerobic energy contributions, thereby mitigating fatigue stimuli such as hypoxia-induced acidosis. Interestingly, absolute VO_2_ peak was the strongest correlate to 19.1 performance and the sole predictor in the model demonstrating that a greater absolute capacity for oxygen consumption rate is an important indicator of performance in 19.1-like WODs compared to the more commonly used measure of VO_2_ max/peak normalized to bodyweight. It is also apparent that fatigue tolerance could be an important factor related to 19.1 performance as participants exert maximal efforts to accomplish as much work as possible within an allotted time. It may be particularly insightful to examine measures of critical power and anaerobic work capacity, such as during a 3 min all-out test, and their relationship to performance in 19.1 or 19.1-like WODs and whether they further impact the predictive model or are superior predictors than VO_2_ max/peak. Recently, Mangine et al. [23] provided some initial insight as critical power determined by a 3 min all-out-test was significantly greater in advanced CF athletes vs. recreational CF participants or physically active subjects.

Previous research with regards to the relationship between strength variables and performance on the non-modified variant of Fran agreed with the present findings on the modified Fran. Butcher et al. [6] found that absolute CF Total was a significant predictor of performance in Fran. In support, subsequent reports by Dexheimer et al. [7] indicated maximum back squat strength (one third of the CF Total measurement) to also be predictive of Fran performance. It is apparent from these studies, including the present, that performance in Fran (or Fran-like WODs) is highly strength-dependent and that those with greater strength, at least measured by CF Total, would perform comparatively well in Fran (modified or non-modified) or Fran-like WODs. One key practical limitation, however, is the lack of overall data to date concerning the relationship between CF Total and Fran to establish normative values. In the present study however, those below the median time-to-completion score (i.e., top performers in modified Fran) had a mean CF Total of 305 kg with the top quartile of performers with a mean of 314 kg. It is anticipated with further data collection, sex-specific normative CF Total values for the prediction of Fran performance can be determined. Also, corresponding with prior studies, measures of aerobic fitness did not correlate with or predict performance in Fran or Fran-like workouts. For instance, according to Bellar et al. [5], aerobic fitness variables, such as VO_2_ max, were not correlated with nor predicted performance in a time-scored workout like Fran in CF-naïve participants. Together, it appears that unlike strength variables, measures reflective of aerobic fitness, such as VO_2_ max/peak and AT) are not indicative of or correlated with Fran or Fran-like workout performance. This is somewhat surprising and in disagreement to previous findings demonstrating the importance of aerobic power in optimal performance of repeated post-anaerobic threshold or maximum workload exercise [24,25,26]. Given that Fran or the present modified Fran requires participants to squat and push a barbell overhead (i.e., thrusters) for a total of 45 repetitions against an absolute resistance while under fatigued conditions (from pull-ups or in the current case, burpees), it is reasonable to suggest that strength variables are predictive of Fran performance. In fact, Martinez-Gomez et al. [27] demonstrated full-squat strength as a determinant of overall CF WOD performance. Individuals with greater total body strength have an increased capacity to squat and press the weight overhead with greater velocity and efficiency leading to a completion of all 45 repetitions in a shorter amount of time and an overall shorter time to complete the entirety of the WOD. Comparatively, strength has also been deemed important for success in activities that require task completion in as little time as possible and performed at or above maximum workload, such as sprint cycling [28,29], further indicating that strength and neuromuscular efficiency, and not necessarily aerobic power, may be more important factors for performance of activities that demand completion of a specific physical loaded task as quickly as possible. Another factor that may explain the strong positive relationship between CF Total and Fran performance is exercise specificity. The CF Total is a standardized CF assessment of total body strength, including the back squat, strict overhead press, and deadlift and likely includes these three specific lifts because they are common movement patterns that are incorporated into CF WODs such as Fran. Thus, there might be an element of movement specificity and specific strength that explains the positive relationship between CF Total and Fran performance. Additionally, given that deadlift, back squat, overhead press 1RM, and CF Total were significantly and strongly correlated with one another, it can be expected that strength in any of the exercises individually would be predictive of Fran performance. The present results provide further verification that total body strength remains an important physiological component of Fran and Fran-like workouts.

The present investigation leads to further questions regarding the physiological indicators of overall CF performance as it appears that individual WODs are explained, at least partially, by distinct determinants of performance. For example, 19.1 performance is not indicated by strength, but rather aerobic fitness while the modified Fran is explained by strength and not aerobic fitness. The results of this study, in addition to those prior, reveal the complexity of competitive CF as each WOD appears to have distinct physiological demands, and thus, training programming to optimize competitive CF performance must be multifaceted to elicit adequate aerobic and strength adaptations. The question remains whether there is an existing testing variable in which scoring would be indicative of overall CF performance (i.e., all the current competition WODs or Open workouts). At present, this does not appear to be the case. It seems that development of multiple physiological and performance variables such as aerobic power and neuromuscular strength is critical for optimum performance in overall competitive CF. In addition, the results of this study should be interpreted with an appreciation of the study limitations. As indicated earlier, the current study was novel in that the influence of CF familiarity on the predictive value of the independent variables was eliminated by recruiting only those naïve to CF. However, this may also present limitations to the practical application of the current findings as it may not apply directly to CF athletes. Thus, it is important to consider the information provided by the current data together with outcomes of prior investigations, in particular the aforementioned studies [5,6,7]. Also, because the present investigation only focused on relationships between predictors and CF performance, our findings do not elucidate the effects of CF training on VO_2_ max/peak, strength, and body composition. Thus, the results should not be interpreted in such context. Further, the WODs implemented during this study are not entirely representative of real-life CF scenarios in which WODs are typically performed in group settings, and real-time modifications are often implemented. The present WODs were performed in an individual setting in order to properly quantify performance while in a controlled testing environment. From a methodology perspective, we recognize the importance of repeated trials to acquire a more substantiated measurement of VO_2_ max or peak [13]. It was decided that a single maximal graded exercise test was the most prudent approach due to logistical constraints of an additional visit, the overall physical stress to the participants, and concerns from the human subjects ethics board concerning participant safety.

In conclusion, performance of two distinct CF workouts were adequately predicted by laboratory-based measurements of fitness which were also distinct and perhaps appropriately specific to the workout. For 19.1, absolute VO_2_ peak was a significant predictor of performance, indicating that aerobic capacity could be a more important characteristic of performance in longer, AMRAP-scored workouts. The CF Total was indicative of performance in a modified Fran, further verifying that maximum strength, at least in the deadlift, overhead press, and back squat, is an important characteristic of optimal performance in Fran and Fran-like workouts (i.e., time-to-completion of exercise tasks against absolute loads). In the context of competitive CF preparations, it remains important for athletes to appreciate and understand the fitness qualities related to success in individual WODs. Athletes may benefit from quantitative aerobic fitness and strength evaluations to guide their training programming and provide knowledge of the factors limiting their performance in AMRAP-style and time-to-completion type WODs. The present results, together with prior findings, reflect the complex nature of competitive CF in that it is a sport comprised of workouts requiring multiple and distinct physiological demands, which in turn makes training for competitive CF an immense challenge compared to other single modal sports.

## Figures and Tables

**Table 1:** Participant characteristics and performance data.

	Total (*n* = 22)	Female (*n* = 9)	Male (*n* = 13)
Descriptive Variables			
Age (year)	22.2 ± 2.9	22.3 ± 3.1	22.2 ± 5.6
Height (cm)	165.4 ± 12.9	157.6 ± 15.7	170.8 ± 7.1
Body Mass (kg)	68.7 ± 9.1	66.4 ± 10.5	70.3 ± 8.2
Body Fat %	19.0 ± 9.8	27.7 ± 8.0	13.0 ± 5.6
Skeletal Muscle Mass (kg)	31.1 ± 5.4	26.3 ± 3.0	34.4 ± 3.8
Metabolic Variables			
Absolute VO_2_ peak (mL/min)	3619.9 ± 870.4	2930 ± 501.0	4097.2 ± 745.0
Relative VO_2_ peak (mL/kg/min)	52.6 ± 9.7	44.2 ± 4.4	58.4 ± 7.9
Absolute VO_2_ @ VT1 (mL/min)	2475.5 ± 636.5	2053.7 ± 377.6	2767.5 ± 622.9
Relative VO_2_ @ VT1 (mL/kg/min)	36.0 ± 7.5	31.0 ± 4.2	39.5 ± 7.4
VT1 (%VO_2_ peak)	68.5 ± 6.3	70.1 ± 6.8	67.3 ± 5.9
Absolute VO_2_ @ VT2 (mL/min)	3295.9 ± 818.1	2726.4 ± 520.8	3690.2 ± 760.8
Relative VO_2_ @ VT2 (mL/kg/min)	48.0 ± 8.9	41.4 ± 4.8	52.5 ± 8.3
VT2 (%VO_2_ peak)	91.0 ± 5.3	92.9 ± 5.8	89.8 ± 4.7
Strength Variables			
Back Squat 1RM (kg)	102.4 ± 29.8	77.3 ± 16.0	119.8 ± 24.1
Overhead Press 1RM (kg)	44.3 ± 15.4	29.8 ± 7.7	54.4 ± 10.4
Deadlift 1RM (kg)	116.3 ± 34.0	85.9 ± 18.6	137.4 ± 24.9
CrossFit^®^ Total (kg)	263.0 ± 76.7	192.9 ± 40.2	311.5 ± 54.5
Relative CrossFit^®^ Total (AU)	3.8 ± 0.9	2.9 ± 0.4	4.4 ± 0.6
CrossFit^®^ Performance Variables			
19.1 Performance (reps)	193.3 ± 32	176.2 ± 27.1	205.2 ± 30.1
Fran Performance (s)	428.8 ± 84.4	465.3 ± 54.4	409.1 ± 92.7

VO_2_ = oxygen consumption rate; VT = ventilatory threshold; RM = repetition maximum. Data presented as mean ± standard deviation.

**Table 2:** Correlations between CrossFit^®^ workout performance and physiological measures.

	19.1 (Reps) (*n* = 22)	Mod. Fran (s) (*n* = 20)
Body Composition		
Body Mass (kg)	0.53 ** p = 0.01	−0.27 *p* = 0.26
Body Fat %	−0.46 * p = 0.03	0.26 *p* = 0.27
Metabolic Variables		
Absolute VO_2_ peak (mL/min)	0.65 ** p = 0.001	−0.35 *p* = 0.13
Relative VO_2_ peak (mL/kg/min)	0.48 * p = 0.02	−0.29 *p* = 0.22
Absolute VO_2_ @ VT1 (mL/min)	0.56 ** p= 0.01	−0.32 *p* = 0.17
Relative VO_2_ @ VT1 (mL/kg/min)	0.36 *p* = 0.10	−0.23 *p* = 0.34
VT1 (%VO_2_ peak)	−0.19 *p* = 0.39	0.02 *p* = 0.92
Absolute VO_2_ @ VT2 (mL/min)	0.61 ** p = 0.002	−0.26 *p* = 0.27
Relative VO_2_ @ VT2 (mL/kg/min)	0.48 * p = 0.03	−0.19 *p* = 0.43
VT2 (%VO_2_ peak)	−0.03 *p* = 0.90	0.35 *p* = 0.13
Strength Variables		
Back Squat 1RM (kg)	0.58 ** p = 0.01	−0.58 ** p = 0.01
Overhead Press 1RM (kg)	0.59 ** p = 0.004	−0.63 ** p = 0.003
Deadlift 1RM (kg)	0.62 ** p = 0.002	−0.57 ** p = 0.01
CrossFit^®^ Total (kg)	0.62 ** p = 0.002	−0.61 ** p = 0.01
Relative CrossFit^®^ Total (AU)	0.46 * p = 0.03	−0.62 ** p = 0.004
CrossFit^®^ Performance Variables		
19.1 Performance (reps)	N/A	−0.50 * p = 0.02
Fran Performance (s)	−0.50 * p = 0.02	N/A

** Correlation is significant at the 0.01 level (2-tailed); * Correlation is significant at the 0.05 level (2-tailed). VO_2_ = oxygen consumption rate; VT = ventilatory threshold; RM = repetition maximum; N/A = not applicable. Data presented as Pearson’s r and *p*-value.

**Table 3:** Summary of multiple regression analysis for 19.1.

Dependent Variable	Independent Variable	B	SE_B_	β	*p*-Value
19.1 Performance	Absolute VO_2_ Peak (mL/min)	0.024	0.007	0.647	0.002

VO_2_ = oxygen consumption rate; B = unstandardized beta; SE_B_ = standard error of B; β = standardized beta.

**Table 4:** Summary of multiple regression analysis for modified Fran.

Dependent Variable	Independent Variable	B	SE_B_	β	*p*-Value
Fran Performance	CrossFit^®^ Total (kg)	−0.667	0.206	0.606	0.005

B = unstandardized beta; SE_B_ = standard error of B; β = standardized beta.
